# Efficacy of Fluorinated Nematicides for Management of Root-knot Nematodes in California Processing Tomatoes

**DOI:** 10.2478/jofnem-2024-0034

**Published:** 2024-10-23

**Authors:** Antoon T. Ploeg, Scott Edwards, Angelo Loffredo, Jörn Ole Becker

**Affiliations:** Department of Nematology, University of California Riverside, 3401 Watkins Drive, Riverside, CA 92521

**Keywords:** fluazaindolizine, fluensulfone, fluopyram, management, *Meloidogyne incognita*, oxamyl, PFAS, root-knot nematodes, *Solanum lycopersicum*, tomato

## Abstract

California is the primary US producer of processing tomatoes. After decades-long excellent protection against the common tropical *Meloidogyne* spp. *M. incognita*, *M. javanica*, and *M. arenaria* (root-knot nematode: RKN) by *Mi*-resistant tomato cultivars, resistance-breaking RKN populations are spreading throughout the San Joaquin Valley. For some time, nematicidal crop management tools have diminished under CA State regulatory pressure. In recent years, new non-volatile compounds have been developed with novel modes of action. In this project with susceptible processing tomato grown on an *M. incognita*-infested site, the efficacy of three fluorinated nematicides was evaluated in 10 field trials from 2011 to 2021. Seven trials included fluensulfone, six included fluazaindolizine, and five included fluopyram. On average, tomato root-galling (0 – 10 scale) at harvest was reduced by 3.2, 2.3, and 2.5 by fluazaindolizine, fluensulfone, and fluopyram, respectively, compared to the untreated control. These reductions in root-galling corresponded with estimated yield gains of approximately 21%, 14%, and 15%, respectively, over the untreated control. The reproduction of RKN during the tomato crop was not affected by the nematicides. The tested compounds have a very low acute mammalian toxicity and are more target-specific than previous generations of nematicides. However, there is considerable concern about the persistence of synthetic per- and polyfluoroalkyl substances (PFAS) in the environment, including pharmaceuticals and pesticides containing fluorine with the C-F3 functional group.

The USA produces about 30% of processing tomatoes (*Solanum lycopersicum*) worldwide, used for tomato pastes, sauces, and canned products. Approximately 91,000 ha were harvested in 2022 in California, which accounts for more than 90% of the US acreage. The total production was 10.5 million tons, valued at $1.1 billion (NASS USDA, 2024). The average yield is approximately 110,000 kg/ha. All processing tomatoes are mechanically harvested when ripe and typically canned within 6 hours after harvest (CTGA, 2024). Root-knot nematodes (RKN: *Meloidogyne* spp.), particularly the Southern root-knot nematode (*M. incognita*) and the Javanese root-knot nematode (*M. javanica*), are economically the most important nematodes of tomato worldwide (Roberts, 1992; Williamson, 1998; Kiewnick et al., 2009). Annual crop losses attributed to RKN in CA processing tomato production at the end of the last century were estimated between 10–20% (Koenning et al., 1999). Since then, there have been many changes in crop management. For RKN management, CA growers mostly rely on two strategies: 1) soil fumigation with metam-sodium, metam-potassium, or 1,3-dichloropropene, which accounted for 19% of the total pesticide active ingredient used in this crop in 2021 (Anonymous, 2021a, 2021b), and 2) nematode-resistance. Increasingly, processing tomato varieties have become available that confer resistance, but not immunity, against the three crucial RKN species *M. incognita*, *M. javanica*, and *M. arenaria* (Roberts, 1992; Williamson, 1998, Lopez-Perez et al., 2006). In 2005, three of the top five cultivars were RKN-resistant (Hartz et al., 2008). Forty-six of the top 50 cultivars were RKN-resistant in 2012 (Miyao et al., 2013), and in 2024, all 92 cultivars in the Processing Tomato Variety Guide were listed as RKN-resistant (TS&L, 2024).

All resistant commercial cultivars share the same single dominant *Mi*-1 gene, which was introgressed into commercial cultivars using embryo rescue from the wild tomato relative *Solanum peruvianum* (Gilbert and McGuire, 1956; Kaloshian et al., 1996; Williamson, 1998). However, resistance-breaking RKN populations occur, and reports of such populations seem to be increasing in tomato-growing areas worldwide (Ploeg et al., 2023). In CA, resistance breaking in tomatoes by RKN was initially reported by Kaloshian et al. (1996), who identified the nematodes as *M. incognita*. Georgia was the second state where tomato resistance-breaking was found (Hajihassani et al., 2022). Ploeg et al. (2023) recently identified 15 resistance-breaking *M. incognita* populations and one *M. javanica* population isolated over two years from processing tomato fields planted with resistant cultivars throughout the Central Valley of CA.

Therefore, an integrated approach where plant resistance is combined or alternated with the use of nematicides may offer growers an effective strategy to slow down and limit the development and build-up of resistance-breaking RKN populations and mitigate damage. The currently used fumigant nematicides carry environmental and health hazards, and their use is being restricted by regulations related to the emission of toxic volatile organic compounds (VOC) that negatively impact air quality (Goodell, 2011; Kroes et al., 2023). Until recently, effective, environmentally acceptable, and economically viable alternatives were not available, and this has been an important factor in the continued use of soil fumigants (Becker, 2014).

In 2011, we initiated a field trial with processing tomatoes to evaluate a novel non-fumigant nematicide, fluensulfone. In 2013, fluazaindolizine, another non-fumigant nematicide, was included for the first time in the trials. A year later, a third non-fumigant nematicide, fluopyram, was tested in two separate field trials. Over an 11-year period, ten field trials with processing tomatoes were done to compare the efficacy of these new nematicides and to test different application rates, methods, and timing. During this period, fluensulfone was registered by the California Department of Pesticide Regulation (CA DPR) for use on fruiting vegetables and root/tuber vegetables. Fluazaindolizine was registered in 2023 by the US Environmental Protection Agency (EPA) for fruiting vegetables, root and tuber crops, some fruit trees, and grapevines, but does not have a CA registration (yet). Fluopyram is registered by CA DPR as a broad-spectrum fungicide and nematicide for suppressing fungal diseases and plant-parasitic nematodes in many annual crops, nut and fruit trees, vines, and turf.

The three tested compounds have major advantages over soil fumigants and previous generations of contact nematicides as they have much lower acute toxicity and carry only a CAUTION label. The precautionary signal word indicates the low potential health risks associated with exposure to the product. Consequently, re-entry intervals are short. There are no buffer zone restrictions or tarping requirements, and worker safety is much improved. Recent publications provide more information about these novel nematicides (Desaeger et al., 2020; Oka, 2020).

The goal of these field trials was to collect information on the efficacy of three non-fumigant nematicides and provide processing tomato growers with alternative options for RKN management.

## Materials and Methods

### Trial site description

Ten field trials were done between 2011 and 2021. The trials were located at the University of California South Coast Research and Extension Center (SCREC), Irvine, CA. The SCREC field was on San Emigdio sandy loam soil (12.5% sand, 75.5% silt, 12% clay, 0.5% organic matter, pH 7.3). The field was infested in 2008 with an egg suspension of an *M. incognita* race 3 population, originally isolated from infested cotton in the San Joaquin Valley, CA. Species and race identifications had originally been done with isoenzyme electrophoresis and on differential host tests (Eisenback and Triantaphyllou, 1991). The population was multiplied on pot-grown tomato plants in a greenhouse at the University of California, Riverside. Eggs were extracted from heavily infested tomato roots by vigorous shaking in a paint shaker (Radewald et al., 2003) for 3 min in 0.5 % NaOCl (Hussey and Barker, 1973) and collected on two 25 μm pore-size sieves. The resulting egg suspension was used to inoculate the fields by injecting it into 4-cm-deep buried drip tubing (Becker et al. 1989). Several *M. incognita*-susceptible vegetable crops were grown sequentially during the spring/summer season to increase and achieve an evenly distributed *M. incognita* infestation level before the trials were initiated. The location of the trials over the infested area was rotated so that the same field area was used for trials once every four years. In between trial years, lima bean, cherry tomato, and okra were grown during the spring/summer season. Hard red winter wheat (*Triticum aestivum* ‘Yecora Rojo’) was grown each winter.

### Trial set-up and treatments

In consecutive years, trials were conducted on different, but nearby areas of the field. Trials I and II were done in 2011, trial III in 2012, trial IV in 2013, trial V and VI in 2014, trial VII in 2015, trial VIII in 2019, and trials IX and X in 2021. In May of each trial year, 102-cm-wide raised beds (center to center) were prepared, and plots were laid out. At the time of preparing the beds, granular fertilizer (N:P:K 15:15:15) at 660 kg/ha and trifluralin herbicide (Treflan EC, Monterey AgResources, Fresno, CA) at 0.56 kg a.i./ha were incorporated. Individual plots were 6-m-long bed sections, separated along the beds by a 91 cm border. The trials were designed as randomized complete blocks with five replicates. An untreated control treatment was included in each trial. The tested nematicides were kindly provided by the manufacturers and included oxamyl (Vydate L, 24% a.i., Corteva Agriscience, Indianapolis, IN), fluensulfone (Nimitz 480 EC, 48% a.i., ADAMA Agricultural Solutions Ltd., Raleigh, NC), fluazaindolizine (Salibro 500 SC, 50% a.i., and a granular formulation 5% a.i., Corteva Agriscience, Indianapolis, IN), and fluopyram (Velum One 400 SC; 40% a.i., Bayer CropScience, Research Triangle Park, NC). All nematicides except the granular formulation of fluazaindolizine were applied in one of two ways: 1) Mixed in 7.6 liters of water and watered evenly over the plot surface with a watering can. An additional 7.6 liters of water was then applied over each plot. All plots were tilled with a rototiller to a depth of 7 to 10 cm. 2) Plots were pre-wetted through 4-cm-deep buried drip tubing (18 mm diameter, emitter output 2 liter/hr, spacing 30.5 cm, Jain Irrigation Inc., Ontario, CA) for 15 min, injected with nematicides through drip tubing for 30 min, and flushed with water for 15 min. The granular fluazaindolizine formulation was applied by evenly distributing the granules over the plots, followed by rototilling and sprinkling 7.6 liters of water on top. The amount of nematicides applied per plot was based on a plot surface area of 3.1 m^2^. An overview of the trials, treatments, rates, application methods, and timing is shown in Table 1.

**Table 1.** Tomato fruit yield and root-galling at harvest as affected by nematicide treatments in ten field trials at the SouthCoast Research and Ex tension Center, Irvine, CA.

**Trial**	**Treatment number**	**Product**	**ai kg/ha**	**Application method^1^**	**Timing^2^**	**kg/plant**	**Galling index**
I	1	untreated control				1.09b^3^	7.1a^3^
	2	oxamyl	0.14	1	5 d pre	1.23ab	5.6b
			0.14	2	21 d post		
	3	fluensulfone	3.84	1	5 d pre	1.48a	3.1c
					*P - value*	0.031	0.002

II	1	untreated control				1.16b	7.0a
	2	oxamyl	0.14	1	5 d pre	1.31ab	6.2a
			0.14	2	21 d post		
	3	fluensulfone	3.84	1	5 d pre	1.42a	5.6ab
	4	fluensulfone	3.84	1	5 d pre	1.23b	3.4b
			3.84	2	21 d post		
					*P - value*	0.015	0.007

III	1	untreated control				1.00	7.7a
	2	oxamyl	0.56	1	at-plant	1.11	7.2ab
			0.56	2	28 d post		
	3	fluensulfone	2.97	1	7 d pre	1.27	6.6b
	4	fluensulfone	4.00	1	7 d pre	1.24	6.4b
					*P - value*	0.12	0.007

IV	1	untreated control				1.18	7.6a
	2	oxamyl	1.12	2	at-plant	1.29	6.2ab
			1.12	2	15 d post		
	3	fluensulfone	1.40	1	7 d pre	1.32	6.5ab
	4	fluensulfone	1.96	1	7 d pre	1.49	4.9bc
	5	fluensulfone	2.81	1	7 d pre	1.37	4.5cd
	6	fluazaindolizine	0.56	2	at-plant	1.47	3.3cd
	7	fluazaindolizine	0.28	2	at-plant	1.41	3.9cd
			0.28	2	15 d post		
	8	fluazaindolizine	0.56	2	at-plant	1.61	2.8d
			0.28	2	15 d post		
					*P - value*	0.06	<0.001

V	1	untreated control				0.74c	9.1a
	2	fluazaindolizine	0.56	2	at-plant	1.23a	2.5de
			0.56	2	14 d post		
			0.56	2	28 d post		
	3	fluazaindolizine	1.12	2	at-plant	1.20a	2.2e
			0.56	2	14 d post		
			0.56	2	28 d post		
	4	fluensulfone	2.97	1	14 d pre	0.91bc	4.2cd
	5	fluensulfone	4.00	1	14 d pre	1.06ab	3.4cde
	6	oxamyl	1.12	2	at-plant	0.95b	6.8ab
			1.12	2	14 d post		
	7	fluopyram	0.25	2	at-plant	1.04ab	4.9bc
			0.25	2	14 d post		
					*P - value*	<0.001	<0.001

VI	1	untreated control				1.05	5.5a
	2	fluazaindolizine	0.56	2	at-plant	1.38	1.5c
			0.56	2	14 d post		
			0.56	2	28 d post		
	3	fluazaindolizine	1.12	2	at-plant	1.43	1.1c
			0.56	2	14 d post		
			0.56	2	28 d post		
	4	fluazaindolizine	1.12	1	3 d pre	1.34	2.4bc
			0.56	2	14 d post		
			0.56	2	28 d post		
	5	oxamyl	1.12	2	at-plant	1.18	5.5a
			1.12	2	14 d post		
			1.12	2	28 d post		
	6	fluopyram	0.25	2	at-plant	1.19	4.3ab
	7	fluopyram	0.25	2	at-plant	1.45	2.2bc
			0.25	2	14 d post		
	8	fluopyram	0.25	2	at-plant	1.34	2.1bc
			0.25	2	28 d post		
					*P - value*	0.21	<0.001

VII	1	untreated control				1.34	5.8a
	2	fluopyram	0.25	2	at-plant	1.62	2.7b
			0.25	2	14 d post		
	3	fluensulfone	2.97	1	14 d pre	1.32	5.8a
	4	fluensulfone	4.00	1	14 d pre	1.44	4.1ab
	5	fluazaindolizine	1.12	2	at-plant	1.61	2.4b
			0.56	2	14 d post		
	6	fluazaindolizine	0.56	2	at-plant	1.51	3.0b
			0.56	2	14 d post		
			0.56	2	28 d post		
	7	fluazaindolizine	2.24	1	3 d pre	1.36	3.7ab
	8	fluazaindolizine^4^	2.24	1	3 d pre	1.52	3.6ab
	9	fluazaindolizine	2.24	1	10 d pre	1.49	3.6ab
	10	fluazaindolizine^4^	2.24	1	10 d pre	1.30	4.1ab
					*P - value*	0.17	<0.001

VIII	1	untreated control				4.07	3.4a
	2	fluensulfone	2.80	1	7 d pre	3.80	1.0b
					*P - value*	0.70	0.009

IX	1	untreated control				2.00	5.6 (a)
	2	fluazaindolizine	1.12	2	at plant	1.98	4.4(ab)
	3	fluazaindolizine	2.24	2	at plant	2.10	2.7(b)
	4	fluazaindolizine	1.12	2	at plant	1.96	3.3(ab)
			0.56	2	30 d post		
	5	fluazaindolizine	1.12	2	at plant	2.04	4.3(ab)
			1.12	2	30 d post		
	6	fluopyram	0.19	2	at plant	2.12	3.7(ab)
					*P - value*	0.99	0.07

X	1	untreated control				2.50	6.0(a)
	2	fluensulfone	2.97	1	7 d pre	2.55	5.6(ab)
	3	fluensulfone	4.00	1	7 d pre	2.80	4.4(ab)
	4	fluopyram	0.19	1	at plant	2.70	5.6(ab)
	5	fluazaindolizine	1.12	1	at plant	2.53	4.1(ab)
	6	fluazaindolizine	2.24	1	at plant	2.66	3.7(b)
					*P - value*	0.99	0.07

1 1: application with watering can in 7.6 L water, followed by 7.6 L water, followed by incorporation. 2: chemigation through buried drip tubing.

2 d pre: days before tomato transplanting, at plant: at tomato transplanting, d post: days after tomato transplanting.

3 different letters within the same trial represent significant differences at the 95% confidence level. When main treatment effects were not significant, lettering is not shown. Lettering in between brackets indicate significant differences at the 90% confidence level.

4 granular formulation, applied by sprinkling over soil followed by incorporation and 7.6 L water per plot.

Tomato transplants were raised from seed in a greenhouse at UC Riverside and planted in the field approximately seven weeks after seeding. Each plot was planted with 20 plants, 30 cm apart, in a single line in the center of the bed. Tomatoes were watered as necessary through buried drip tubing, and in-season fertilization was according to local standard practices. In trials I-VII, the tomato cultivar was Halley 3315 VFF (OrsettiSeed Company, Hollister, CA) and in trials VIII-X tomato U373 VF (Nunhems USA, Parma, ID).

### Data collection

To determine initial pre-treatment (Pi) and final at-harvest (Pf) RKN levels, a composite sample consisting of six cores of soil (1.5 cm diameter, from a depth between 5 and 30 cm) was collected from each plot just before applying the first treatment and just before harvest. Nematodes were extracted from 100 g soil subsamples placed on Baermann funnels for 5 days at 26°C (Southey, 1970). Second-stage juveniles (J2) of RKN were counted at x40 magnification.

In each trial, all fruits were harvested by hand from all plots once and at the same time when about 70% of tomatoes were ripe, approximately 4 months after transplanting. The total fruit weight per plot was determined. The root systems of five plants per plot were dug and rinsed with water. The severity of galling on the five root systems was visually assessed, and a galling index was assigned from 0 to 10, where 0 = no galls and 10 = 100% of roots galled (Zeck, 1971). The average of the galling indices of the five root systems was used as the plot galling index.

### Data analysis

The statistical significance of the treatment effects on the soil nematode multiplication factor (at-harvest J2 per 100 g soil/initial J2 per 100 g soil) and yield (kg fruit per plant) was analyzed using an analysis of variance (ANOVA) procedure with blocks considered as random effects. Post-hoc separation of treatment means was done using Fisher’s protected least significant difference (LSD) test (*P* ≤ 0.05). Before analysis, the soil nematode multiplication factor was transformed by x^1^= log_10_(x+1). The assumption of normality of the log_10_[(Pf/Pi)+1] and yield (kg/plant) data was tested with the Shapiro-Wilk test. Treatment effects on tomato root-galling were analyzed using the non-parametric Kruskal-Wallis test also at the 95% confidence level.

To determine if a decrease in root-galling resulting from the nematicide treatments was correlated with an increase in tomato yield, the difference between the average root-galling index in the untreated control and in each treatment was calculated for each trial according to: gall_diff_ = gall_utc_ − gall_trt_. The percentage yield difference of each treatment relative to the untreated control was also calculated for each trial according to: yield_diff_ = 100*yield_trt_/yield_utc_ − 100. The data were plotted with gall_diff_ as the predictor variable on the *x*-axis and yield_diff_ as the response variable on the *y*-axis. Polynomial regression models were fitted through the data with degrees of *h* = 1 to 5. Analysis of mean standard errors of the five models showed that a quadratic model (*h*=2) best fitted the data. R software (R Core Team, 2022) was used for statistical analysis and regression model fitting.

## Results

The initial RKN levels (Pi) ranged between 1 and 110 J2/100g soil for trial VIII treatment 2 and trial V treatment 7, respectively, and at-harvest RKN levels (Pf) between 9 and 2,477 J2/100g soil for trial VIII treatment 2 and trial I treatment 1, respectively. The soil J2 multiplication factor ranged from 2.2 to 74.2 in trial VI treatment 2 and trial III treatment 1 (Table 1), respectively. Statistical analysis of the soil J2 multiplication factor showed that log_10_[(Pf/Pi)+1]-transformed data were normally distributed in each trial (Shapiro-Wilk test, *P* > 0.05). The soil J2 multiplication factor was never significantly different among the treatments (*P* > 0.05; data not shown).

The yield data (kg fruit per plant) were also normally distributed in each trial (Shapiro-Wilk test, *P* > 0.05), and the treatments significantly affected the yield in only three of the ten trials (Table 1). In trials I, II, and V, a single pre-plant fluensulfone application at 3.84–4 kg ai/ha increased yields compared with the untreated control. In trial V, oxamyl, fluazaindolizine, and fluopyram treatments also resulted in significantly higher tomato yields than the untreated control (Table 1).

Treatment effects on tomato root-galling were more common, with eight trials showing significant effects of the treatments on galling. In the remaining two trials (IX and X), treatment *P*-values were 0.07 (Table 1). The untreated control treatment resulted in the highest galling index in each trial. However, in two trials (VI and VII), one of the nematicide treatments resulted in an equally high galling index.

The first three trials focused on comparing the efficacy of fluensulfone with oxamyl. In trial I, a repeated application of oxamyl and a single pre-plant application of fluensulfone both reduced root-galling compared with the untreated control, but fluensulfone was more effective. Furthermore, fluensulfone increased yields, whereas oxamyl did not. In trials II and III, the oxamyl treatments did not significantly reduce galling or improve yields, but three of four fluensulfone treatments were effective in reducing root-galling (Table 1).

In trial IV, fluazaindolizine was evaluated for the first time and compared to oxamyl and fluensulfone. In this trial, the two higher rates of pre-plant applied fluensulfone (1.96 and 2.81 kg/ha), and all three fluazaindolizine treatments (once 0.56 kg/ha, twice 0.28 kg/ha, 0.56 kg/ha followed by 0.28 kg/ha) significantly reduced galling. Oxamyl was not effective (Table 1).

Fluopyram was first included as a treatment in trial V, which also included oxamyl, fluensulfone, and fluazaindolizine. Oxamyl was the only nematicide treatment that failed to reduce galling. The two fluazaindolizine treatments resulted in strong and significant reductions in galling and also resulted in the highest yield increase compared to the untreated control treatment. The fluopyram treatment also reduced galling but not as effectively as fluazaindolizine. The efficacy of the two fluensulfone treatments in this trial was intermediate between fluazaindolizine and fluopyram.

In trial VI, three treatments of fluazaindolizine and fluopyram were compared to oxamyl. The latter again failed to significantly reduce galling, and the fluazaindolizine treatments again were very effective in reducing root-galling. The fluopyram treatments were also effective, particularly when applied as a split application of two times 0.25 kg/ha (Table 1). Because of the generally poor performance of oxamyl in trials I-VI, it was no longer included as a treatment in subsequent trials.

All three fluorinated nematicides were included as treatments in trial VII, with an emphasis on fluazaindolizine, which was applied in different formulations, at different rates, and as a single or split application resulting in a total of six fluazaindolizine treatments. The four single pre-plant soil-incorporated applications of the liquid or granular formulation at 2.26 kg/ha (treatments 7–10, Table 1) did not significantly reduce galling. However, the two drip-tubing-applied split applications (treatments 5 and 6, Table 1) significantly reduced galling. Fluopyram was again effective. The two fluensulfone treatments did not result in significantly lower root-galling compared with the untreated control.

In trial VIII, only a single pre-plant incorporated fluensulfone treatment was included. Although the average at-harvest root-galling was low even in the untreated control (galling index 3.4), the fluensulfone treatment still significantly reduced galling to a very low level (Table 1).

Treatment effects on root-galling in trials IX and X, both done in 2021, were not significant at the 95% confidence level (treatment effect on root-galling: *P* = 0.07 in both trials). However, in both trials, each nematicide treatment resulted in numerically lower root-galling levels than the untreated control (Table 1).

Rates of single pre-plant fluensulfone applications in the trials ranged between 1.4 and 4.0 kg/ha. In trial IV, where rates of 1.4, 1.96, and 2.81 were compared, the lower rate did not significantly reduce galling compared to the untreated control. It was less effective than the two equally effective higher rates which did significantly reduce galling. Direct comparisons between fluensulfone rates of 2.97 and 4.00 kg/ha were included in four trials (III, V, VII, X). There were no significant differences in efficacy to reduce root-galling between these two rates in any of these four trials (Table 1). Following a pre-plant application of fluensulfone with a post-plant application was evaluated only once, in trial II. This treatment is, however, not allowed according to the US EPA label. Although this treatment reduced root-galling compared with the untreated control, it also significantly reduced yields compared with a one-time pre-plant fluensulfone application (Table 1).

Total amounts of fluazaindolizine applied in the trials ranged from 0.56 to 2.24 kg/ha and were applied as a one-time pre- or at-plant treatment or as at-plant followed by post-plant treatments. The lowest rate of 0.56 kg/ha was only used in trial IV in two treatments: a one-time at-plant treatment (trial IV treatment 6) or as a split application (twice 0.28 kg/ha, trial IV treatment 7). Even at these low rates, the two treatments were very effective in reducing root-galling (Table 1). A comparison between total amounts of 1.68 kg/ha and 2.24 kg/ha in split applications was included in trials V, VI, and IX. These treatments effectively reduced galling in trials V and VI but not in trial IX. However, there were no indications that the higher rate was more effective in reducing root-galling (Table 1). A granular formulation of fluazaindolizine was compared with the liquid formulation in trial VII at 2.24 kg/ha applied 3- or 10-days pre-plant. These treatments did not significantly reduce root-galling compared with the untreated control and were not different from each other (Table 1, trial VII treatments 7–10).

Total amounts of fluopyram applied in the trials were 0.19, 0.25, or 0.5 kg/ha. The lower rate was applied in trials IX and X, and in both these trials, there were no significant treatment effects (Table 1). The higher rate was only applied as a split application (at-plant and post-plant) of two times 0.25 kg/ha and significantly reduced root-galling in all three trials where it was included as a treatment (Table 1, trial V treatment 7, trial VI treatments 7 and 8, trial VII treatment 2). A single at-plant fluopyram treatment at 0.25 kg/ha, however, did not significantly reduce root-galling compared with the untreated control (Table 1, trial VI treatment 6).

**Figure 1.**
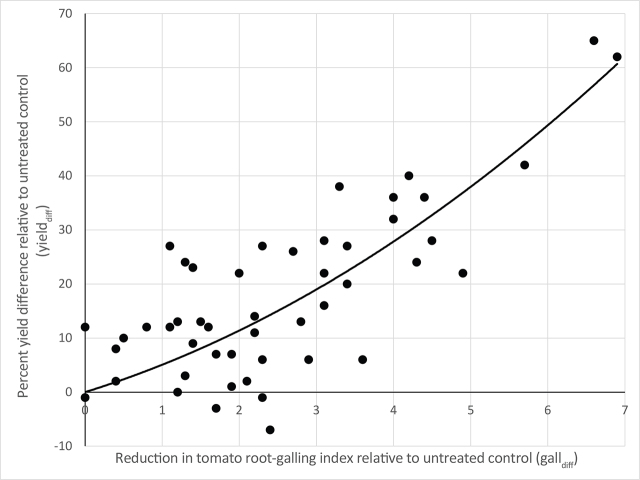
Correlation between reduction in tomato root-galling (gall_diff_) and percent yield difference (yield_diff_) relative to untreated control treatments from ten field trials. Line fitted according yield_diff_ = 0.63 × gall_diff_^2^ + 4.43 × gall_diff_. adjusted R^2^=0.83.

Regression analysis of the difference in average tomato root-galling between untreated controls and nematicide treatments in each trial (gall_diff_) and the percentage yield difference of the nematicide treatments relative to the untreated controls in each trial (yield_diff_) showed that the quadratic model:

$${\text{yield}}_{\text{diff}}=0.63\times {{\text{gall}}_{\text{diff}}}^{2}+4.43\times {\text{gall}}_{\text{diff}}$$

best fitted the data (adjusted R^2^ = 0.83, Figure 1). Averaged over all trials, fluensulfone reduced the galling index compared to the untreated control with 2.3, fluazaindolizine with 3.2, and fluopyram with 2.5. According to this model, achieving these reductions in root-galling corresponds with yield gains of 14%, 21%, and 15%, respectively, over the untreated control.

## Discussion

The use of *Mi*-resistant tomato cultivars has been the foundation of RKN management in California for many years. Populations of RKN able to overcome this resistance were rare in the past (Kaloshian et al., 1996) but have become more common in several major CA production areas (Ploeg et al., 2018). At the same time, back-to-back tomato planting in the same location on beds with semi-permanent deep-buried drip irrigation tubing is practiced. Winter cropping with wheat, which generally exhibits only minor RKN root symptoms but sustains the nematodes (Martin et al., 2021), may further enhance the survival of RKN in the absence of tomato.

The CA IPM Center at UC Davis identified two critical research issues on RKN management in processing tomatoes: 1) the development of cultivars with new sources of RKN resistance and 2) the evaluation of new nematicides (Martin et al., 2021). Although other RKN resistance genes have been identified in wild tomato germplasm (Veremis and Roberts, 1996; Ammiraju et al., 2003), they have not yet been incorporated into new tomato cultivars.

The development of fluorinated nematicides, a class of new contact nematicides, and, perhaps more importantly, active ingredients with novel modes of action has significantly changed our standard operating procedure (Desaeger et al., 2020; Oka, 2020). In our studies, results from ten years of field trials with susceptible processing tomato on a site infested with medium to high levels of an *M. incognita* race 3 RKN population confirmed the utility of the three tested nematicides as they reduced root-galling and improved yield.

The three tested nematicides have much improved toxicological profiles and are generally considered more benign to non-target organisms than previous carbamate and organophosphate nematicides. All three products showed excellent efficacy in initial pot trials (Loffredo et al., 2024), but their performance in the field was much more variable, and their field application presented some challenges. Fluensulfone can result in delayed germination and/or reduced vigor in direct-seeded crops such as carrots (Becker et al., 2019), cucumbers (Ploeg, unpublished data), and lima beans (Jones et al., 2017) when applied at or close to seeding. However, phytotoxic effects were not observed in any of the trials in this study with transplanted tomatoes, where fluensulfone applications ranged between 14 days pre-plant and 21 days post-plant. Fluopyram is, in vitro, an excellent nematicide at very low concentrations (Faske and Hurd, 2015), second only to abamectin. However, Grabau et al. (2021), in a review comparing the efficacy of the three fluorinated nematicides from lab, greenhouse, and field tests, reported that fluopyram was not consistently better than the other two. Fluopyram has low soil mobility (Becker et al., 2019; Faske and Brown, 2019; Rathod et al., 2022), and consequently, the activity does not extend much beyond the application zone. This effect was clearly demonstrated in carrot trials where only the upper part of the carrot roots remained free of galling after a shallow soil-incorporated application of fluopyram (Becker et al., 2019), whereas fluensulfone and fluazaindolizine protected the entire carrot roots from galling. The significant galling on the lower part of the carrot roots after a fluopyram application still rendered them unmarketable, resulting in fluopyram being much less effective in preventing a loss in marketable carrots compared to fluensulfone and fluazaindolizine (Becker et al., 2019). Whereas a carrot with a root-galling index of > 3 (0 – 10 scale) is considered unmarketable (Becker et al., 2019), in tomatoes, there is no such cut-off value for root-galling resulting in a complete crop loss. Consequently, in these trials, no major differences in the efficacy among the three fluorinated nematicides were observed.

In vitro tests with fluazaindolizine showed that nematode mortality occurred only after several days of exposure (Thoden and Wiles, 2019; Wu et al., 2020) but also that sublethal concentrations still reduced or inhibited nematode motility and infectivity (Wram and Zasada, 2019). Averaged over all trials, fluazaindolizine was the most efficacious product, reducing tomato root-galling by 3.2 compared to the untreated controls, which corresponds with a predicted yield increase of 21% over the untreated controls. A similar result was obtained in carrot field trials with the same three fluorinated nematicides, where fluazaindolizine was also the most effective product (Becker et al., 2019).

Oxamyl is still registered in California for nematode management in tomatoes, but it did not significantly reduce tomato root-galling compared to the untreated control in five out of the six trials that included an oxamyl treatment. This lack of efficacy has been observed previously, particularly in warm, moist, alkaline soils with elevated soil pH, which correlated with a short persistence of the nematicide (Haydock et al., 2012).

The fluorinated nematicides tested in these trials were effective in reducing root-galling in tomatoes. They could be used where resistance-breaking populations occur or in an integrated system with susceptible cultivars to avoid damage and selection of resistance-breaking populations. In trial IV, the high rate of fluensulfone reduced galling more than the low rate. In general, however, there were no differences between rates or application methods of the nematicides.

Recently, polyfluoroalkyl substances (PFAS) have become under scrutiny because of potential environmental and health concerns. These synthetic chemicals are highly stable and have a high attraction to lipids. This could result in bioaccumulation and ecotoxicological effects in soils and water environments (Alexandrino et al., 2022). All three fluorinated nematicides in this study are included in a list of PFAS pesticides published by the Minnesota Department of Agriculture (https://www.mda.state.mn.us/environment-sustainability/active-inert-pfas). The European Union has proposed a wide-ranging ban on fluorinated compounds (Rossen, 2023), and several US states have planned similar restrictions on PFAS-containing pesticides. The compounds are categorized as long-, short, and ultra-short-chain PFAS, which have >7, 4–7, and 2–3 fully fluorinated C-atoms, respectively (Wang et al., 2017). While most studies have focused on long-chain PFAS, uncertainty remains concerning the toxicity of ultra-short and short-chain PFASs, especially regarding long-term effects, emphasizing the need for further research (Ateia et al., 2019; Fenton et al., 2021). Thus, the future of the fluorinated nematicides used in our studies remains uncertain, but banning the whole spectrum of PFAS, which includes more than 12,000 compounds, would have many unexpected consequences, including impacting an increasing number of pharmaceutical and plant protection compounds (Spyrakis and Dragani, 2023; Tyrrell, 2023).
